# Rapid Bacterial Detection in Urine Using Laser Scattering and Deep Learning Analysis

**DOI:** 10.1128/spectrum.01769-21

**Published:** 2022-03-02

**Authors:** Kwang Seob Lee, Hyung Jae Lim, Kyungnam Kim, Yeon-Gyeong Park, Jae-Woo Yoo, Dongeun Yong

**Affiliations:** a Department of Laboratory Medicine, Yonsei University College of Medicine, Seoul, South Korea; b Department of Research and Development, The Wave Talk, Inc., Daejeon, South Korea; c Research Institute of Bacterial Resistance, Yonsei University College of Medicine, Seoul, South Korea; University of California, San Diego

**Keywords:** deep learning, laser scatter, prediction, urinary tract infection, rapid tests

## Abstract

Images of laser scattering patterns generated by bacteria in urine are promising resources for deep learning. However, floating bacteria in urine produce dynamic scattering patterns and require deep learning of spatial and temporal features. We hypothesized that bacteria with variable bacterial densities and different Gram staining reactions would generate different speckle images. After deep learning of speckle patterns generated by various densities of bacteria in artificial urine, we validated the model in an independent set of clinical urine samples in a tertiary hospital. Even at a low bacterial density cutoff (1,000 CFU/mL), the model achieved a predictive accuracy of 90.9% for positive urine culture. At a cutoff of 50,000 CFU/mL, it showed a better accuracy of 98.5%. The model achieved satisfactory accuracy at both cutoff levels for predicting the Gram staining reaction. Considering only 30 min of analysis, our method appears as a new screening tool for predicting the presence of bacteria before urine culture.

**IMPORTANCE** This study performed deep learning of multiple laser scattering patterns by the bacteria in urine to predict positive urine culture. Conventional urine analyzers have limited performance in identifying bacteria in urine. This novel method showed a satisfactory accuracy taking only 30 min of analysis without conventional urine culture. It was also developed to predict the Gram staining reaction of the bacteria. It can be used as a standalone screening tool for urinary tract infection.

## INTRODUCTION

Urinary tract infections (UTIs) are a common cause of sepsis in health care-associated infections and the most common infections among outpatient clinics (1). UTIs include pathogenic infections at anatomical sites from the urethra to the kidney (2). In a multinational study, the focus of infection was associated with the urogenital system in 30% of sepsis patients, and 12% of nosocomial UTI patients developed urosepsis (3). Inappropriate antibiotic therapy or late management is associated with a higher mortality in the patients with urosepsis (4, 5). Therefore, immediate administration of broad-spectrum antibiotics after urine culture is crucial (1). Therefore, when physicians are suspicious of a UTI that requires further antibiotic treatment, prompt reporting of whether the patient is in a true infection state should be performed. Furthermore, accurate pathogen identification is necessary to guide physicians regarding narrow-spectrum antibiotic usage. Currently, the standard method for identifying uropathogens is urine culture. The urine culture process is used to quantitatively report the pathogen by spreading 1 μL or 10 μL of the urine sample onto the culture plates. For complete isolation and identification of the pathogen, this process takes 24 to 48 h (6). In clinical settings, several urine analyzers equipped with flow cytometry or automated microscopy are used to rapidly screen for the presence of urinary bacteria in patients. The urine analyzer offers a screening method to determine whether urine culture or further testing of the infection status is needed (7–10). However, the use of urine analyzers for classifying the Gram stain characteristics of bacteria has not yet been validated.

**FIG 1:**
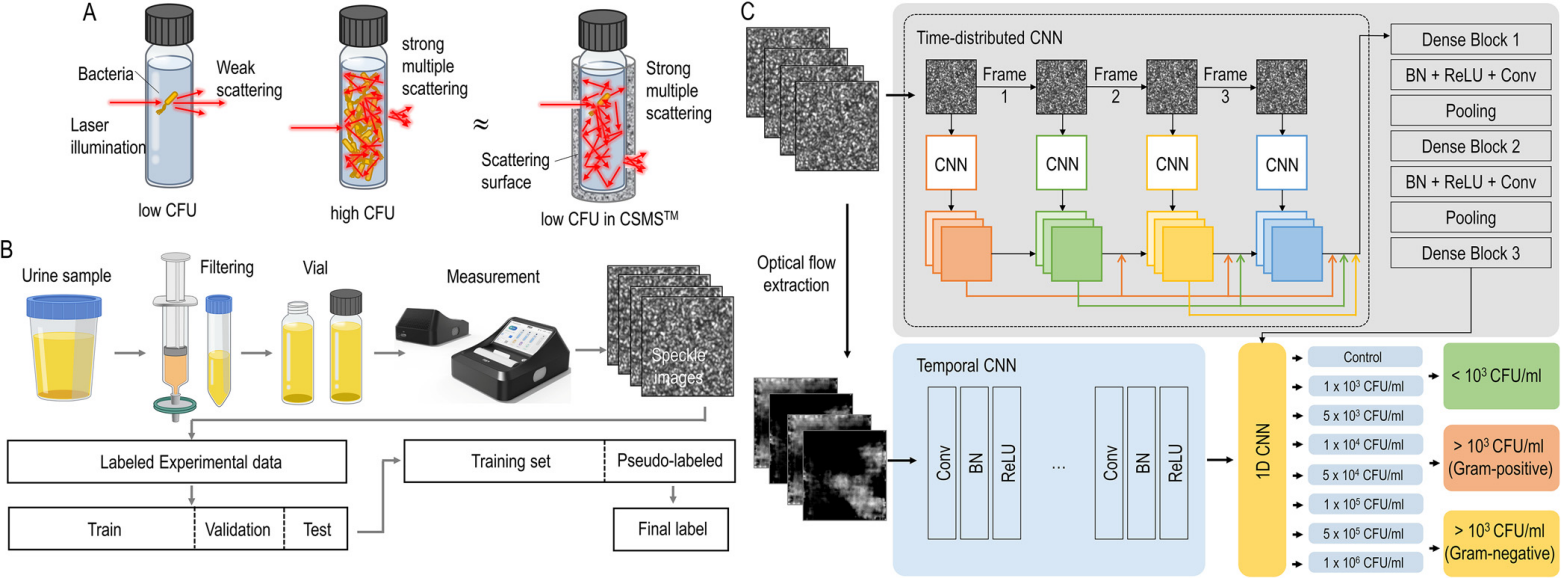
Schematic of “The Wave Talk” sensor system. (A) Multiple reflections of light increase the chance of light-bacterium interaction, which works as an amplification signal. (B) Schematic diagrams of urinary tract infection diagnosis and data processing for deep learning architecture. (C) Proposed two-stream network architecture. CNN, convolutional neural network.

To overcome the disadvantage of the urine analyzer and the culture, optical sensing and machine learning have been applied in rapid diagnostics. One research study used machine learning to train the peptide signatures by mass spectrometry from uncultured urine to rapidly identify bacterial species (11). Another research study used deep learning of single bacterial cell spectra to identify bacterial species without culture (12). Here, we constructed a system combining optical sensing technology and machine learning, named as the Bacometer. The Bacometer system exploits multiple light scattering to maximize the interaction between the light and the target bacteria (Fig. 1A), which results in highly enhanced scattering signals from the bacteria and the formation of time-varying speckle patterns.

In this article, we validated this novel method for the prediction of positive urine culture and provide a Gram staining result using deep learning analysis of the laser scattering patterns of bacteria in urine.

## RESULTS

### Patient characteristics.

We constructed a prediction model by training the Bacometer using the laser scattering patterns of nine American Type Culture Collection (ATCC) strains diluted with artificial urine (see Table S1 in the supplemental material). The trained model was clinically validated using 263 clinical urine samples from the testing set (Table 1). The positive culture rate was 29.7% (78/263), and the possible contamination rate of the specimens was 3.8% (10/263), both of which were significantly higher in females (chi-square test: culture rate, *P* value = 0.009; contamination rate, *P* value = 0.006). To compare the accuracies of the two assays, 215 samples that simultaneously underwent random urinalysis were investigated. The levels of nitrite, leukocyte esterase, bacteria, and white blood cells were used as surrogate markers of positive urine cultures. The prediction rate of urinalysis for positive urine culture was 42.8% (92/215), whereas 44.6% (including possibly contaminated specimens) among them were true positives according to bacterial culture (Table 1).

**TABLE 1:** Characteristics and demographics of patients^*a*^

Characteristic	Total no.	No. (%) with culture result:		
		Positive	Negative	Possible contamination
Male	147	35 (23.8)	111 (75.5)	1 (0.7)
Female	116	43 (37.1)	64 (55.2)	9 (7.7)
Age (yr) (IQR)	60.0	60.5 (46.3–70.8)	59.0 (40.5–69.0)	56.5 (50.5–71.5)
Inpatient	190	54 (28.4)	130 (68.4)	6 (3.2)
Outpatient	73	24 (32.9)	45 (61.6)	4 (5.5)
Urinalysis result (positive/total)	92/215	37 (40.2)	51 (55.4)	4 (4.4)
Nitrite	11	9 (81.8)	2 (18.2)	0 (0)
Leukocyte esterase	62	29 (46.8)	29 (46.8)	4 (6.4)
Bacteria (>25/HPF)	14	11 (78.6)	3 (21.4)	0 (0)
WBC (>2/HPF)	79	33 (41.8)	43 (54.4)	3 (3.8)

a Samples with results that stated “quality not satisfied” in any of the four predictive parameters or samples in which urinalysis was not performed within the same day as or the day before urine culture were excluded from analysis. Possible contamination was defined as having three or more isolates without predominance in a culture. IQR, interquartile range; HPF, high-power field; WBC, white blood cell.

### Clinical performance validation of the Bacometer.

Of the 263 clinical urine samples, 215 paired automated microscopic urinalysis results were analyzed to compare performances between the two assays. Urinalysis exhibited a low sensitivity (55.4%) and specificity (63.8%), whereas the Bacometer produced more accurate results, with a sensitivity of 75.7% and specificity of 97.9% when the cutoff level for positive urine culture was set at 1,000 CFU/mL (Table 2, *P* value < 0.05).

**TABLE 2:** Performance of urinalysis and the Bacometer using paired clinical urine samples (*n* = 215) with 95% confidence interval for each parameter^*a*^

Parameter	Urinalysis	Bacometer^*b*^	*P* value
Sensitivity (%)	55.4 (44.1–66.7)	75.7 (65.9–85.5)	0.007
Specificity (%)	63.8 (55.9–71.8)	97.9 (95.5–100.0)	<0.001
PPV (%)	44.6 (34.4–54.7)	94.9 (89.3–100.0)	<0.001
NPV (%)	73.2 (65.3–81.0)	88.5 (83.4–93.5)	<0.001
Accuracy (%)	60.9 (54.1–67.5)	90.2 (85.5–93.9)	NA
TP/FP/TN/FN (no.)	41/51/90/33	56/3/138/18	

a PPV, positive predictive value; NPV, negative predictive value; TP, true positive; FP, false positive; TN, true negative; FN, false negative; NA, not applicable.

b The cutoff level for positive urine culture prediction by the Bacometer was set at ≥1,000 CFU/mL.

**FIG 2:**
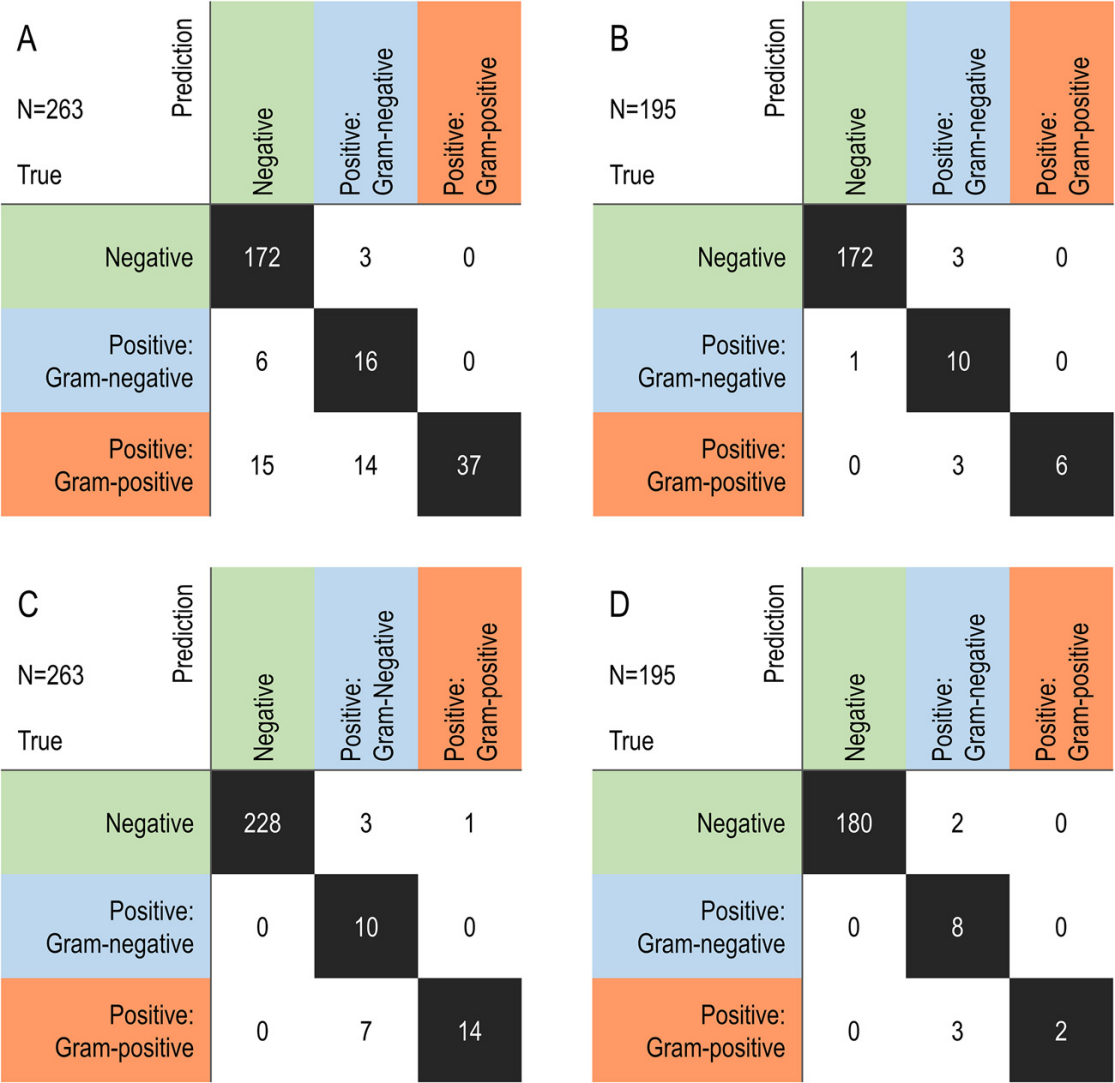
Confusion matrix describing the predictive performance of Gram staining reaction with different cutoff levels for the prediction of positive urine culture. (A) Samples overall, cutoff ≥1,000 CFU/mL. (B) Trained-species samples, cutoff ≥1,000 CFU/mL. (C) Samples overall, cutoff ≥50,000 CFU/mL. (D) Trained-species samples, cutoff ≥50,000 CFU/mL.

The advantage of this novel assay is the direct prediction of the Gram staining reaction in the collected urine samples without colony isolation. Therefore, the Bacometer was evaluated for its predictive ability with respect to the Gram staining reaction results of the predicted positive urine culture samples. The evaluation was divided into two subsets: the first set incorporated all collected samples (*n* = 263) and the second contained those with positive cultures for pathogens of the trained species (*n* = 195) (Table 3; Fig. 2). Furthermore, the performance of the assay was evaluated at two different cutoff levels for positive culture prediction (1,000 CFU/mL and 50,000 CFU/mL). At the 1,000 CFU/mL cutoff level, the predictive accuracy was 90.9%, with a sensitivity of 76.1% and specificity of 98.3%. For the prediction of the Gram staining reaction among the predicted positive urine culture samples, the accuracy was 79.1% (Table 3; Fig. 2A). For the second subset (*n* = 195), the derived parameters improved, with an accuracy of 97.9%, sensitivity of 95.0%, and specificity of 98.3%, which produced an accuracy of 84.2% for the prediction of the Gram staining reaction (Table 3; Fig. 2B).

**TABLE 3:** Performance parameters for the prediction of positive urine culture in two subsets (overall and trained-species set) according to the two cutoff values for positive urine culture

Testing set (no.)	Cutoff (CFU/mL)	Accuracy (%)	Sensitivity (%)	Specificity (%)
Overall (263)	1,000	90.9	76.1	98.3
	50,000	98.5	100.0	98.3

Trained species (195)	1,000	97.9	95.0	98.3
	50,000	99.0	100.0	98.9

When the cutoff level for positive culture was set at 50,000 CFU/mL, the predictive accuracy was 98.5%, with a sensitivity of 100.0% and specificity of 98.3%. Among the predicted positive urine culture samples, the predictive accuracy for the Gram staining reaction was 77.4% (Table 3; Fig. 2C). For the second subset, the predictive accuracy for positive culture improved to 99.0%, whereas the accuracy for the Gram staining reaction was 76.9% (Table 3; Fig. 2D).

### Distribution of pathogens.

Among the true-positive (*n* = 67) and false-negative (*n* = 21) samples identified by the Bacometer, we determined the distribution of the pathogens identified from the plate culture (see Table S2). Gram-positive cocci and Escherichia coli were the most frequent pathogens in true-positive samples, which is consistent with previously published data (13, 14). All false-negative samples were found to have a bacterial density of <10,000 CFU/mL.

## DISCUSSION

Conventional methods for urine culture differ from those for other specimens (e.g., blood and sputum). Bacterial load was reported using a semiquantitative method that provides grown CFU per milliliter of urine using calibrated loops. Although 100,000 CFU/mL is widely accepted as the decision level for UTI (15), clinical laboratories report positive urine culture results using a cutoff level of 1,000 to 10,000 CFU/mL, considering the patient’s status or collection method. For instance, urine samples obtained by catheterization are considered positive for 1,000 to 50,000 CFU/mL of bacteria (16).

Meanwhile, it is a great burden for laboratories to streak every urine sample onto culture plates, while clinical blood cultures are performed only in culture bottles that are alarmed with a positive signal. Therefore, it is important to adopt screening tools or provide precise alternative methods for urine culture to control the workload in laboratories.

In this study, we validated a novel and rapid bacterial screening method using a convolutional neural network. The clinical validation set consisted of samples that were chosen randomly on a routine working day. The prevalence of positive urine cultures and the distribution of isolated pathogens in the validation set guaranteed the absence of sampling bias (see Table S2 in the supplemental material).

Since conventional light-scattering techniques use only single-scattered light from a specimen, the scattering signals from bacterial solutions with low concentrations are too low to be detected owing to the low scattering cross sections. This is inevitable because of the microscopic size of individual bacteria and small refractive index contrast between the bacterial cytoplasm and surrounding medium (17). Conventionally, bacterial culture is required to visually detect the presence of bacteria. In a sample with a high concentration of bacteria, the interactions of light and bacteria significantly increase due to multiple light scattering, which is analogous to highly diffusive fog or clouds consisting of transparent microscopic water droplets (18). The principle of the Bacometer is to generate multiple light scattering using a scattering surface without time-consuming bacterial culture. Even at a very low concentration of bacteria, the light incident to a sample containing a scattering surface undergoes multiple interactions due to multiple light scattering, resulting in strong scattering signals equivalent to samples with a high concentration of bacteria. As a result, the Bacometer system can detect the scattering signals from individual bacteria with a significantly enhanced signal-to-noise ratio. This principle can be understood as a significant amplification of an optical path length without physically increasing a detection volume at a given sample concentration.

The use of multiple light scattering to enhance optical signals has been utilized to overcome the sensitivity limitations of conventional optical devices (18), including scattering super lenses (19), temperature sensors (20), pressure sensors (21), wavelength meters (22), fiber-based spectrometers (23), absorption spectrometers (24), and nonresonant lasers (25).

Previous attempts have been made to use optical sensing technology for rapid identification of bacteria (26). Recently, extensive deep learning of bacterial Raman spectra has been used to identify 30 common pathogens and their antibiotic susceptibility, with an overall accuracy of approximately 82% (27). Other methods using optical scattering technology have been applied for rapid identification of solid colonies of Campylobacter spp. and common UTI pathogens (28, 29). Currently, the U.S. FDA-approved laser scattering methodology for the qualitative detection of bacteriuria is the BacterioScan 216Dx UTI system (30, 31). According to a recent study, this system predicts UTI at a cutoff level of 50,000 CFU/mL, with a sensitivity of 92.1%, specificity of 82.7%, positive predictive value (PPV) of 44.8%, and negative predictive value (NPV) of 98.6% (30). The turnaround time of the system was 3 h.

Our optical method expedites the detection of Gram-positive or Gram-negative pathogens by exploiting multiple scattered laser signals from bacteria with analysis using deep learning. Using a convolutional neural network, deep learning of amplified laser scattering patterns refracted by bacteria enabled the determination of the presence of the pathogen at 1,000 CFU/mL in uncultured urine and facilitated the determination of the Gram staining reaction of the pathogen. Clinical validation of the method showed a superior accuracy compared to conventional urinalysis, and the method showed enhanced performance with respect to the classification of samples that were later discovered to be one of the pathogens used for training the system. Due to the high NPV and PPV, the Bacometer would suffice as a standalone screening tool for further specimen processing. Furthermore, among the clinical samples with nine pathogens used for deep learning training, the predictability was satisfactory with respect to the Gram staining results.

A limitation of this study is that polymicrobial infections could not be filtered out through the current system until conventional culture was performed. Deep learning of laser scattering patterns of polymicrobial samples may solve this issue in the future. Second, although most of the samples with yeast in the urine were correctly predicted for positive urine culture, the system still needs to be trained with a number of species. Considering that the system was trained using only nine species, the deep learning of more scattering patterns of diverse species (e.g., *Candida* and other Streptococcus spp.) will improve the performance of the system. Furthermore, a prospective study of the clinical impact of the Bacometer should be performed.

In conclusion, the Bacometer demonstrated reliable clinical performance through a simple 30-min processing of urine specimens. Prediction of the Gram staining reaction of the pathogen and the infection status at the lower limit of bacterial density could provide faster and more appropriate interventions for UTI patients.

## MATERIALS AND METHODS

### Bacometer.

The Bacometer system consists of a sensor unit that generates multiple scattering with a diode-pumped solid-state coherent laser (λ = 532 nm, 20 mW) as a light source and a complementary metal-oxide semiconductor (CMOS) sensor (Fig. 1A). The sensor unit has an aluminum structure that can generate multiple light scattering so that the incident light can be scattered and reflected several times inside, maximizing light-matter interaction.

The light emitted from the sensor unit forms a specific speckle image generated by the interference of the different light paths produced via multiple scattering and the use of a temporally coherent light source. A camera is located at the output port of the scattering surface, which has an offset to the input port of the scattering surface to minimize unscattered light signals at the camera plane. The speckle pattern is measured using a CMOS sensor. The sensor is located so it captures each speckle grain size, corresponding to approximately 2 by 2 pixels (32–36).

### Training of the Bacometer using ATCC strains.

Nine strains were obtained from the ATCC. The bacteria used in all experiments were grown in nutrient broth (NB) and Luria-Bertani (LB) broth. The bacterial suspension was centrifuged at 6,000 rpm (3,663 × *g*, 4°C) for 10 min to obtain a pellet. Bacterial cell pellets were washed with phosphate-buffered saline (1× PBS) in the same tube. UTI-causing strains were cultured using MacConkey (BD Biosciences, Franklin Lakes, NJ, USA) medium and Columbia CNA (BD Biosciences, Franklin Lakes, NJ, USA) medium with 5% sheep blood and incubated at 37°C for 20 h. Escherichia coli (ATCC 25922) concentration was adjusted to an optical density at 600 nm (OD_600_) of 1.0 to 1.2 (10^8^ CFU/mL). Other strains were adjusted using the same method (see Table S1 in the supplemental material). This study used eight concentration intervals (control [artificial urine], 1 × 10^3^, 5 × 10^3^, 1 × 10^4^, 5 × 10^4^, 1 × 10^5^, 5 × 10^5^, and 1 × 10^6^) of bacteria initially diluted with artificial urine at a 1:100 ratio. This process was repeated eight times for each strain. The artificial urine used in this experiment (artificial urine medium, 1700-0018; Pickering; lot.010058) was manufactured with a composition similar to human urine and suitable for clinical research and product testing. Artificial urine can also grow a wide range of urinary pathogens and form crystals found in actual urinary tract infections.

### Deep learning.

We used new neural network architectures inspired by classification techniques for human action recognition, which can process both the spatial and temporal information contained in videos (multiframe image data). Dynamic speckle patterns were analyzed using a DenseNet-based neural network to distinguish the presence of pathogens at a concentration of 1,000 CFU/mL in uncultured urine and the Gram staining characteristics of the pathogen. A “two-stream network” incorporates two streams of data (Fig. 1C), the spatial and temporal streams. The spatial stream processes sequential speckle image frames and comprises a time-aligned DenseNet network (37) that feeds 300 frames from each sample. The temporal network was also trained to receive optical flow data comprising grayscale heatmaps, highlighting the movement of bacteria between two sequential frames in a speckle image. A single data sample fed into the temporal network consisted of data describing the interframe optical flow from a series of two frames, with a separate frame for movements in the vertical and horizontal planes, resulting in a 2-channel input image of 256 by 256 pixels in a chunk. The temporal network was encapsulated in a time-distributed layer, followed by a one-dimensional global average pooling layer, allowing 100 sets of 3-frame chinks within the speckle images to be processed. Finally, the spatial and temporal global average pooling layers were concatenated before the final output layer. This design is inspired by the two-stream networks pioneered by Feichtenhofer et al. (38) but differs in several key aspects. Most notably, our temporal stream comprised an untrained DenseNet model. Unlike Feichtenhofer’s implementation, this network contained no fully connected layers and used the global average pooling of feature maps, which resulted in significantly fewer trainable parameters, leading to fast convergence with improved accuracy. Each network was trained until the validation loss had plateaued. The models were saved after each epoch, and the model with the highest validation accuracy was used for the final assessment of the test set. The final output layer of each network comprised three densely connected neurons. The loss was calculated using the categorical focal loss function, and the weights were updated using the Adam optimizer. The batch sizes for all the networks were 10. Programming was performed with the Python programming language using TensorFlow (39) and Keras (40) machine learning frameworks and the Keras-vis package (41).

### Procedures for data acquisition and algorithm training.

The data acquisition and algorithm training procedures are summarized in Fig. 1B. From the experimental data acquired with the Bacometer, the data for the training set were split into 86% training (57,600 images) and 14% validation (9,600 images) groups. A total of 9,600 images of the validation data were obtained through additional experiments to enhance the robustness of the deep learning model in practical UTI diagnosis. Samples for the validation data were manufactured using the same procedure as that used for obtaining the training data, except that a healthy person’s random urine was used. The training data set contained 7,200 images of the control (artificial urine) sample and 50,400 images of seven concentration spike samples. The data from the validation set were then used to train the model. We trained the models for 500 epochs using the Adam optimizer in the nonprivate setting and the stochastic gradient descent (SGD) optimizer in the private setting. We preevaluated our framework using an eight-concentration class classification task. The test set was predicted, and the results were tagged using pseudolabels. The whole data (training and validation) were split to ensure validation and to use to train the model using the focal loss function. Finally, by using the pseudolabels, the original test set was predicted for model evaluation, which was formulated as a three-class classification (negative, positive [Gram positive], and positive [Gram negative]).

### Sample collection and conventional (reference) method.

Routine clinical urine samples were analyzed in the clinical microbiology laboratory of a tertiary hospital (Severance Hospital, Seoul, South Korea). Gram staining and urine culture were performed and interpreted by trained medical technicians and confirmed by medical laboratory staff. For urine culture, 1 μL of urine was inoculated on 5% blood agar and MacConkey agar plates (Asan Pharmaceutical, Seoul, South Korea). The plates were then incubated for 24 h in a 5% CO_2_ incubator at 37°C. The interpretation of the culture depends on CFU per mL. A plate showing <1,000 CFU/mL (no visible colonies) was considered a negative culture result. Otherwise, plates were considered positive results and processed for bacterial identification using conventional methods, including the Vitek system (bioMérieux, Marcy l’Etoile, France) and/or matrix-assisted laser desorption ionization–time of flight mass spectrometry (Vitek MS; bioMérieux Inc., Durham, NC). Possible contamination of urine samples was defined as follows, which was modified from the clinical procedure guideline: (i) ≥3 bacterial isolates and (ii) without predominant isolates (<10^5^ CFU/mL) (42). This study was approved by the Institutional Review Board of Yonsei University (IRB no. 4-2020-0021).

### Preparation of the clinical urine samples.

After collecting >4 mL of urine using a needleless syringe, the urine was passed through a filter (pore size, 5 μm; diameter, 25 mm; Tisch Scientific, North Bend, OH). The pore size was chosen to shift host-derived cells and particulates, considering the size of the bacterial cells (<5 μm). Filtered urine (2.27 mL) was then injected into the Bacometer.

### Urinalysis.

Urinalysis was performed using an Atellica 1500 automated urinalysis system (Siemens Healthineers, Eschborn, Germany). The prediction of positive urine culture in the urine samples was considered positive if one of the following criteria was met: (i) bacteria of >25/high-power field (HPF), (ii) nitrite positive, (iii) white blood cell count of >2/HPF, and (iv) leukocyte esterase equal to or more than a trace. The diagnostic performance of routine urinalysis was compared with the results of the conventional culture method.

### Data analysis.

To validate the Bacometer with clinical samples, urinalysis and culture results were retrospectively reviewed. The reference label for positive urine culture was defined as the growth of any organism in the urine culture, including possible contaminated samples. If multiple species were isolated in the urine culture, the reference label for the Gram staining reaction was defined as the most abundant isolate in the culture. To compare the sensitivity and specificity values for predicting positive urine culture, McNemar’s test was performed between the urinalysis and the Bacometer. For PPV and NPV comparison, the generalized score statistic method was used (43). Confidence intervals were derived using the Wald formula.

### Data availability.

Patient data were stored on a server at the Severance Hospital, South Korea. The model training data and the clinical feature extracted data are available from the corresponding author upon request and subject to ethical review.
