# Supplementary material for: Rapid Bacterial Detection in Urine Using Laser Scattering and Deep Learning Analysis
Source: Microbiol Spectr. 2022 Mar 2;10(2):e01769-21. doi: 10.1128/spectrum.01769-21 (PMC8941854; doi:10.1128/spectrum.01769-21)
Supplement: SUPPLEMENTAL FILE 1 — Supplemental material. Download SPECTRUM01769-21_Supp_1_seq4.pdf, PDF file, 0.1 MB [file spectrum01769-21_supp_1_seq4.pdf]

Supplementary Table 1. Profiles of the reference bacterial strains tested in the experiment.

Strains	ATCC No.	Medium	Gram-stain	OD_{600}
Escherichia coli	ATCC25922	NB	Negative	0.10~0.12
Klebsiella pneumoniae	ATCC700603	NB	Negative	0.10~0.12
Proteus mirabilis	ATCC35659	NB	Negative	0.08~0.09
Pseudomonas aeruginosa	ATCC27853	NB	Negative	0.22~0.24
Staphylococcus aureus	ATCC29213	LB	Positive	0.27~0.28
Staphylococcus epidermidis	ATCC12228	LB	Positive	0.33~0.35
Staphylococcus saprophyticus	ATCC15305	LB	Positive	0.38~0.40
Streptococcus agalactiae	ATCC13813	LB	Positive	0.30~0.33
Enterococcus faecalis	ATCC29212	LB	Positive	0.21~0.23

ATCC, American Type Culture Collection; OD_{600}, optical density at 600 nm; NB, nutrient broth; LB, Luria-Bertani broth

Supplementary Table 2. Positive predicted samples by the Bacometer at a cut-off level of 1,000 CFU/ml and the distribution of uropathogens isolated from the reference bacterial culture method.

Isolated pathogen	Number	CFU/ml		
True positives by the Bacometer	67	1,000-10,000	10,000-50,000	≥50,000
Gram-positive cocci	14	11	3	
Escherichia coli	6			6
Gram-negative rods	5	4		1
Enterococcus faecium	4		3	1
Gram-positive rods	4	1	1	2
Staphylococcus spp.	4	3	1	
Candida albicans	3			3
Diphtheroids	3		2	1
Enterococcus faecalis	3			3
Klebsiella pneumoniae	3			3
Lactobacillus spp.	3		1	2
Candida glabrata	2	1	1	
Candida tropicalis	2		1	1
α-*Streptococcus*	2		1	1
Carnobacterium spp.	1			1
Candida parapsilosis	1			1
Candida utilis	1			1
Citrobacter spp.	1			1
Corynebacterium spp.	1			1
Proteus mirabilis	1		1	
Staphylococcus aureus	1			1
CoNS	1			1
Yeast	1		1	
False negatives by the Bacometer	21	1,000-10,000	10,000-50,000	≥50,000
Gram-positive cocci	11	11		
Gram-negative rods	5	5		
Gram-positive rods	2	2		
Enterococcus faecium	1	1		
Escherichia coli	1	1		
Yeast	1	1		

CoNS, coagulase-negative Staphylococcus; isolated bacteria reported as gram-positive cocci, gram-negative rods and gram-positive rods were samples with minimal reporting protocol referred to the laboratory policy and clinical guideline (1), therefore, certain species were not given.

Supplementary reference

1. Chan W. 2016. Urine Cultures. *In* Leber AL (ed), Clinical microbiology procedures handbook, 4th ed, ASM Press, Washington, DC, https://doi.org/10.1128/9781555818814.ch3.12.
